# A Novel Pathogenic Variant Identified in HIKESHI-Related Hypomyelinating Leukodystrophy Disrupts Heat Shock Response in iPSCs

**DOI:** 10.3390/ijms26136037

**Published:** 2025-06-24

**Authors:** Mahmood Ali Saleh, Maria Boichuck, Aner Ottolenghi, Tatiana Rabinski, Omri Goldenthal, Daniel Sevilla Sanchez, Aviva Fattal-Valevski, Gali Heimer, Shay Ben-Shachar, Stephanie Libzon, Orly Gershoni-Yahalom, Anat Ben-Zvi, Raz Zarivach, Ayelet Zerem, Benyamin Rosental, Gad David Vatine

**Affiliations:** 1 The Department of Physiology and Cell Biology, Faculty of Health Sciences, Ben-Gurion University of the Negev, Beer Sheva 8410501, Israel; alisaleh@post.bgu.ac.il (M.A.S.); rabinski@bgu.ac.il (T.R.); goldenth@post.bgu.ac.il (O.G.); 2 The Regenerative Medicine and Stem Cell (RMSC) Research Center, Ben-Gurion University of the Negev, Beer Sheva 8410501, Israel; mariaboi@post.bgu.ac.il (M.B.); anerster@gmail.com (A.O.); orlyge@bgu.ac.il (O.G.-Y.); 3 The Shraga Segal Department of Microbiology, Immunology and Genetics, Faculty of Health Sciences, Ben-Gurion University of the Negev, Beer Sheva 8410501, Israel; 4 Ilse Katz Institute for Nanoscale Science and Technology, Ben-Gurion University of the Negev, Beer Sheva 8410501, Israel; sevillasanchez.daniel@gmail.com; 5 Pediatric Neurology Institute, Dana-Dwek Children’s Hospital, Tel-Aviv Sourasky Medical Center, Tel-Aviv 6423906, Israel; aviva.fatal@gmail.com (A.F.-V.); steph.libzon@gmail.com (S.L.); ayeletze@tlvmc.gov.il (A.Z.); 6 Faculty of Medicine and health sciences, Tel Aviv University, Tel-Aviv 6997801, Israel; gali.heimer@sheba.health.gov.il (G.H.); shayb@clalit.org.il (S.B.-S.); 7 Pediatric Neurology Unit, Safra Children’s Hospital, The Sheba Medical Center, Ramat Gan 5262000, Israel; 8 Clalit Research Institute, Ramat-Gan 5262000, Israel; 9 Department of Life Sciences, National Institute for Biotechnology in the Negev, Ben-Gurion University of the Negev, Beer Sheva 8410501, Israel; anatbz@bgu.ac.il (A.B.-Z.); zarivach@bgu.ac.il (R.Z.); 10 Department of Life Sciences, Ilse Katz Institute for Nanoscale Science and Technology, Ben-Gurion University of the Negev, Beer Sheva 8410501, Israel; 11 The Zelman Center of Neuroscience, Ben-Gurion University of the Negev, Beer Sheva 8410501, Israel

**Keywords:** HIKESHI-related hypomyelinating leukodystrophy (HHL), hypomyelination, heat shock (HS), nuclear translocation, HSP70, iPS cells

## Abstract

HIKESHI-related hypomyelinating leukodystrophy (HHL) is a life-threatening disorder caused by homozygous pathogenic variants in *HIKESHI*. Symptoms include infantile onset progressive spastic dystonic quadriplegia, nystagmus, failure to thrive, diffused hypomyelination, and severe morbidity or death following febrile illness. V54L variants in *HIKESHI* are particularly prevalent within the Ashkenazi Jewish population. Here, we identified a novel P78S disease-causing variant in *HIKESHI* in a patient of Christian Arab origin, presenting with clinical and radiologic features characteristic of HHL. In silico analysis suggests that the mutated residue may affect the HIKESHI protein’s dimerization domain. We generated a comprehensive set of induced pluripotent stem cells (iPSCs) from the index case and two additional HHL patients. To investigate mechanisms potentially linked to febrile illness in HHL, we used these cells to study the heat shock (HS) response. HHL-iPSCs showed dramatically decreased levels of HIKESHI compared with healthy controls following HS. In addition, they exhibited increased *HSP70* mRNA levels in response to HS, suggesting an increased sensitivity. HHL-iPSCs had impaired HSP70 translocation to the nucleus. Our results provide a human-relevant model for HHL.

## 1. Introduction

HIKESHI-related hypomyelinating leukodystrophy (HHL) is a rare and devastating disorder characterized by an infantile onset of failure to thrive, axial hypotonia, progressive spastic dystonic quadriplegia, nystagmus, and acute deterioration and death during febrile illness. In most cases, the perinatal history is unremarkable. Typically, the first symptoms appear between 3 and 10 months of age, including feeding difficulties, hypotonia, and delayed motor development. Nystagmus may also be observed. Over time, hypotonia progresses to a complex motor disorder characterized by spasticity, dystonia, and ataxia. During early childhood, children often acquire motor and language milestones such as sitting, crawling, and producing words or simple sentences. Progression of spasticity and deterioration in motor abilities are commonly observed during adolescence [1,2]. The prognosis of HHL patients remains poor, with no targeted therapeutic options. HHL is caused by autosomal recessive inactivating mutations in the *HIKESHI* gene, also known as *C11ORF73*. A recurrent homozygous missense variant, c.160G>C, p.(Val54Leu) in *HIKESHI*, has been linked to HHL, particularly prevalent within the Ashkenazi Jewish (AJ) population, with an estimated heterozygous carrier frequency of approximately 1 in 200 [1]. An additional variant c.11G>C, p.(Cys4Ser) was identified in a single Finnish patient [3], and more recently, another variant c.4T>G, p.Phe2Val was reported in a single patient of Afghan descent [4], both exhibiting similar HHL symptoms.

Severe morbidity or death during febrile illness were reported in HHL patients, and 8 out of 16 described patients died suddenly at a mean age of 7.5 years (range: 1–15 years) in a clinical setting of cardiogenic shock following febrile illness. The reported body temperatures during these episodes ranged from 38 °C to 41.5 °C. While HHL patients may experience febrile illnesses without severe attacks, fever in these cases typically remains low. The current data are insufficient to define a temperature threshold for life-threatening events, and caution is warranted during any febrile episode. Life expectancy is uncertain, with the oldest reported patient reaching 29 years. These findings suggest that increased sensitivity to heat shock (HS) response may underlie this disorder [1,2,3]. Interestingly, HIKESHI is known to operate as a nuclear import carrier that facilitates shuttling of the molecular chaperon Heat Shock Protein 70 (HSP70) into the nucleus during HS stress [5], where it plays a crucial role in protecting cells against heat stress-induced protein misfolding and aggregation [6]. Thus, HSP70 translocation to the nucleus is imperative for the restoration of normal cellular conditions and cell viability. Dysfunctional HIKESHI may therefore impair the translocation of HSP70 into the nucleus during HS, resulting in compromised reversal of protein misfolding and, consequently, leading to HS-induced nuclear phenotypes [2,6,7,8,9].

Here, we report a novel pathogenic variant c.232C>T, p.(Pro78Ser) in a newly identified HHL patient. We generated and fully characterized induced pluripotent stem cells (iPSCs) from two patients with a homozygous V54L and the P78S HHL patient, along with their sex-matched healthy heterozygous family relatives. Our findings revealed that iPSCs from both V54L and P78S patients exhibited HIKESHI depletion and very low levels, respectively, resulting in impaired HSP70 translocation into the nucleus following HS. This suggests a potential role for HIKESHI in development and lays the groundwork for future examination of its involvement in disease-relevant lineages, such as neural cells and cardiac tissue.

## 2. Results

### 2.1. Identification of a Novel Homozygous Pathogenic Variant in an HHL Patient

**Figure 1:**
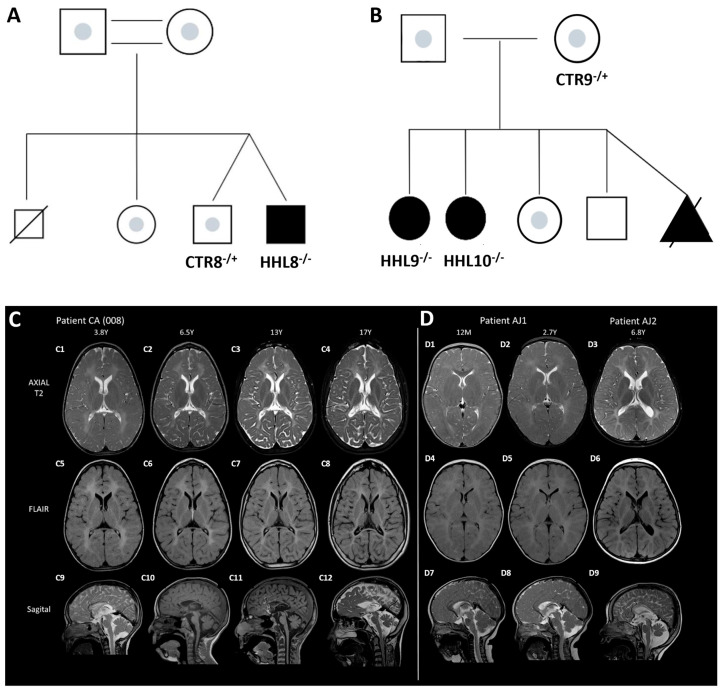
Family pedigrees and brain MRI. Family pedigrees describing (**A**) the index case (P78S) and (**B**) the V54L HHL families. Squares and circles denote male and female sexes, respectively. Homozygous HHL patients are marked with filled black shapes, and heterozygous carriers are marked with an inner gray spot. Consanguineous marriage is marked by two parallel lines. The black triangle describes a pregnancy loss of a homozygous fetus. A line across the square describes a deceased individual with an unknown genotype. (**C**,**D**) Brain MRI of HHL patients. The ages of the patients are listed at the top of each column. Sequential brain MRI scans of the index case (**C1**–**C12**): Diffuse and confluent T2 signal hyperintensities of the entire supratentorial white matter consistent with diffuse hypomyelination (**C1**–**C8**). Over time, gradual loss of white matter involving both gray and white matter that is more pronounced posteriorly (**C2**–**C4**,**C6**–**C8**). Thin corpus callosum more dominantly in the posterior part with gradual atrophy including brainstem and cerebellum (**C9**–**C12**). Brain MRI scans of two AJ HHL patients at corresponding ages (**D1**–**D9**): Diffuse and confluent T2 signal hyperintensities of the entire supratentorial white matter consistent with diffuse hypomyelination (**D1**–**D6**) and at later age white matter volume loss (**D3**,**D6**). Thinning of corpus callosum more prominent posteriorly (**D7**–**D9**). Brainstem and cerebellar atrophy in later age (**D9**).

The proband is a 21-year-old male of CA origin presenting with severe progressive spastic dystonic quadriplegia, intellectual disability, anarthria, nystagmus, short stature, acquired microcephaly, and epilepsy. Trio Exome sequencing at the age of 16.5 years identified a homozygous variant: c.232C>T Pro78Ser (p.P78S) in the *HIKESHI* gene, inherited from both carrier parents (Figure 1). A detailed case report description is provided in the Supplementary Data.

Repeated brain magnetic resonance imaging (MRI) tests were conducted. The first MRI, performed at 10.5 months of age, revealed delayed myelination and a thin corpus callosum. Follow-up MRIs showed diffuse hypomyelination involving the supratentorial and infratentorial white matter. Gradual loss of white matter and thinning of the corpus callosum were observed at later ages. Further progression in volume loss, involving both gray and white matter, was noted and was more pronounced posteriorly, involving infratentorial structures including the brainstem and cerebellum. These clinical and radiologic features of the index case were comparable with HHL patients carrying the V54L mutation that were previously reported [1] (Figure 1 and Supplementary Data).

### 2.2. In Silico Analysis of the HIKESHI Protein Reveals That the Pathogenic Variants Are Located Within a Hydrophobic Pocket

**Figure 2:**
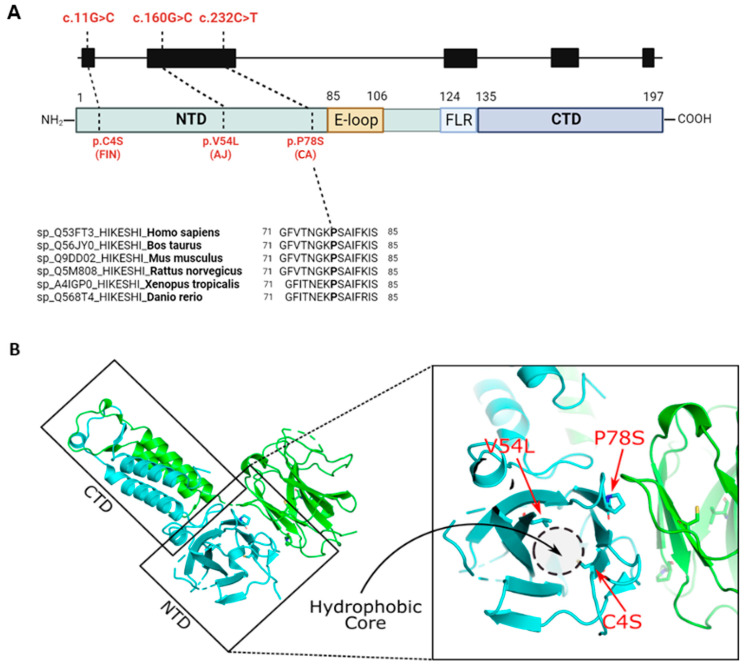
Conservation and localization of the P78S pathogenic variant. (**A**) Schematic description of the HIKESHI gene and protein. The three different bi-allelic pathogenic variants in HHL patients are represented including the founder AJ variant c.160G>C, p.V54L, the (c.11G4C, p.C4S) mutation that was recently reported in a Finnish patient (FIN), and the novel (c.232C>T, p.P78S) mutation that was identified in a CA family. The P78 residue is well conserved in vertebrate evolution (lower panel). N-terminal domain (NTD). Extended loop (E-loop). Flexible linker region (FLR). C-terminal domain (CTD). (**B**) HIKESHI protein 3D analysis revealed that the V54 and C4 that were associated with the AJ and Finn mutations, respectively, were located within a hydrophobic pocket. The P78 residue that was associated with the CA mutation was located within the dimerization region. Left panel, a dimeric HIKESHI structure (PDB: 3WVZ) [10] in ribbon representation colored green and cyan per monomer. Right panel, zoom-in on the N-terminal domain (NTD) hydrophobic core region. The locations of the three pathogenic mutations are depicted in red. The figure was made with PyMol (PyMOL Molecular Graphics System, Version 2.0 Schrödinger, LLC, New York, NY, USA).

The P78 residue is well conserved throughout vertebrate evolution and is located within the protein N-terminal domain, in proximity to the extended loop (E-loop), which was previously suggested to cover the hydrophobic pocket [10] (Figure 2A). To evaluate the pathogenic effect of the P78S mutation, we analyzed the three-dimensional (3D) structure of the HIKESHI protein (PDB: 3WVZ) [10] using the PyMOL molecular visualization system in which we denoted the location of the two known and the novel pathogenic variants (Figure 2). Both the V54L and C4S mutations were located within the hydrophobic pocket. The V54L mutation was expected to cause an enlargement of the residue by a methyl group. Consequently, the L54 residue could potentially collide with the V38 residue resulting in changes in the packing (formation) of the hydrophobic core. The C4S would replace a hydrophobic residue of the hydrophobic cysteine with the polar serine, which would most likely prevent it from folding into a hydrophobic pocket and might cause alterations in the protein folding. These observations were in agreement with previously published data [3]. The newly identified P78S mutation was localized to the predicted dimerization region in the hydrophobic loop [10]. Proline could play a crucial role in breaking the beta-sheet into a loop structure within the protein. A mutation to serine at this position had the potential to alter the folding of the protein and consequently impact the overall protein landscape. Moreover, proline was predicted to engage in hydrophobic interactions with L10. Altering proline to serine may lead to the break of the dimeric interface. Since HIKESHI functions as a homodimer, such a mutation could potentially impact both sides of the dimeric interface. 

### 2.3. Generation of Patient-Specific iPSCs from HHL Patients and Healthy Controls

**Figure 3:**
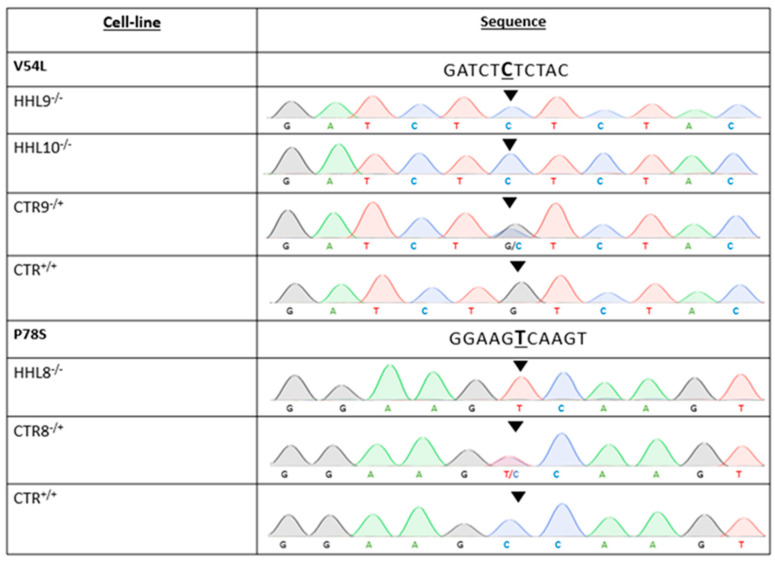
iPSC genotype (related to Supplementary Figures S1 and S2). DNA sequencing of two families with HLL including the AJ family with homozygous (HHL9^−/−^ and HHL10^−/−^) or heterozygous (CTR9^−/+^) c.160G>C mutations (top panel) as well as the unrelated control (CTR12^+/+^), and the CA family with homozygous (HHL8^−/−^) or heterozygous (CTR8^−/+^) c.232C>T mutation, as well as the unrelated control (CTR^+/+^, bottom panel).

To obtain biological samples from HHL patients, we collected skin punch biopsies from the HHL patient carrying a homozygous c.232C>T, p.(P78S) variant and from two HHL patients with a homozygous c.160G>C, p.(V54L) variant in HIKESHI. In addition, we collected cells from their healthy sex-matched heterozygous family relatives and from an additional, unrelated non-carrier healthy individual to serve as the control (CTR). Biopsies were harvested to generate skin fibroblasts, which were then reprogramed to iPSCs using non-integrating episomal vectors as previously described [11,12,13,14]. All iPSCs were fully characterized for the expression of pluripotency markers and their ability to differentiate into the three germ layers, had a normal karyotype, and were mycoplasma free. Finally, we isolated genomic DNA and sequenced the variant locus from all generated lines to validate the genotypes (Figure 3 and Supplementary Figures S1 and S2).

### 2.4. HIKESHI Levels Are Significantly Decreased in HHL iPSCs Following Heat Shock

**Figure 4:**
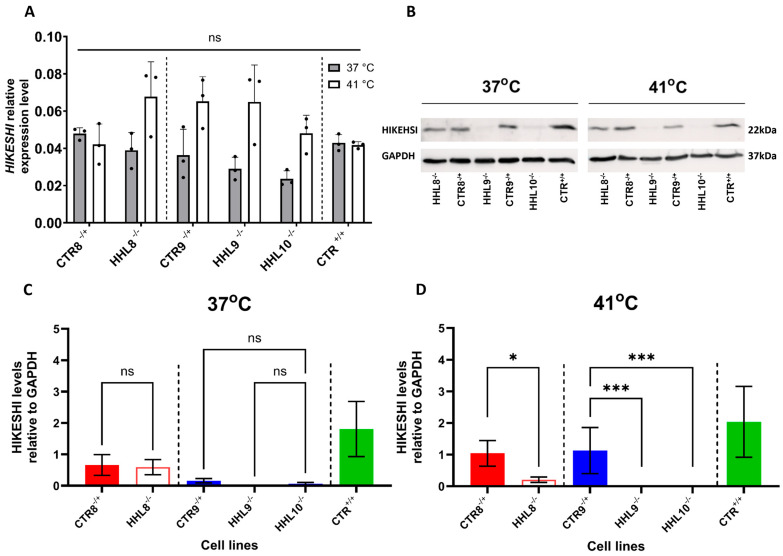
HIKESHI mRNA and protein levels in response to HS (related to Supplementary Figure S3). (**A**) HIKESHI relative mRNA expression in iPSCs from P78S (HHL8^−/−^) and V54L (HHL9^−/−^, HHL10^−/−^) patients and from CTRs (CTR8^−/+^, CTR9^−/+^, and CTR12^+/+^). RNA was extracted from iPSCs incubated under normal (37 °C) or HS (41 °C) conditions for a period of 4 h. The HIKESHI transcript level was determined by qRT-PCR and normalized to levels of the housekeeping gene GAPDH. Data are presented as mean ± SEM of three different passages using a single clone from each iPSC line, each performed in triplicates. Each data set was tested for normal distribution followed by multiple unpaired *t*-test. * *p* < 0.05, *** *p* < 0.001. (**B**) Representative blots of HIKESHI and GAPDH in P78S and V54L families under normal (37 °C, left panel) or HS (41 °C, right panel) conditions. Quantification of HIKESHI protein levels of undifferentiated iPSCs cultured at 37 °C (**C**) or following four hours of HS at 41 °C (**D**) for all lines normalized to GAPDH. Data represent nine independent experiments performed at different passages on at least two clones from each line. Mann–Whitney analysis was conducted for comparison of each patient line and their corresponding CTR from the same family.

Severe morbidity or death during febrile illness were reported in HHL patients, suggesting that increased sensitivity to HS may underlie this disorder [2]. To investigate their response to febrile stress, we cultured iPSCs from HHL patients and CTRs either under standard culture temperature (37 °C) or under an elevated temperature (41 °C), representing a febrile illness relevant temperature, for a 4 h duration to simulate HS. We observed comparable levels of HIKESHI mRNA in both HHL and CTR cell lines at 37 °C. Following HS treatment, there was a trend toward an increase in HIKESHI mRNA levels in some of the lines, but these changes were not statistically significant (Figure 4A). These results suggested that HIKESHI mRNA levels were not affected by HS and that the pathogenic variants likely did not alter HIKESHI transcriptional regulation. We next tested HIKESHI protein expression by Western blot analysis (Figure 4B–D). HIKESHI levels in CTR cells were not significantly affected by HS (Figure 4C,D). Interestingly, the V54L-HHL cells were completely depleted from HIKESHI, and the P78S-HHL had nearly undetectable HIKESHI levels following HS. These results suggested that pathogenic HIKESHI variants were degraded at the protein level.

### 2.5. Impaired HSP70 Localization Following HS in HHL iPSCs

**Figure 5:**
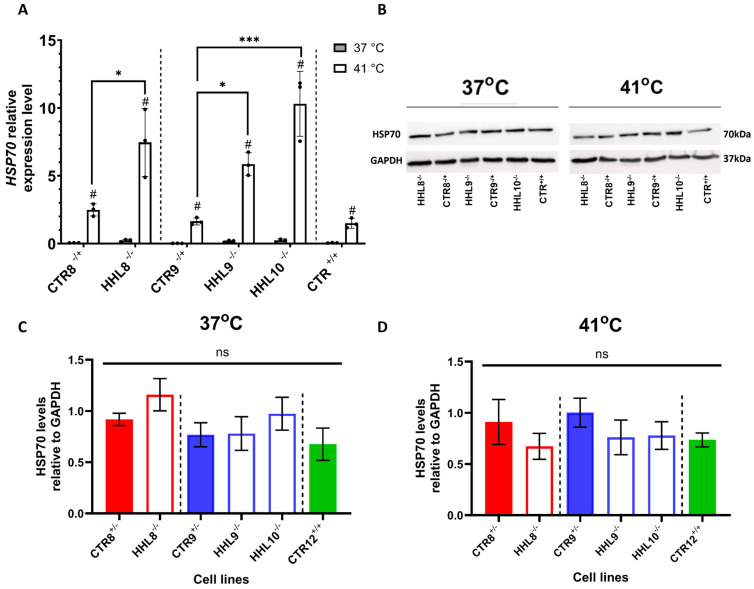
HSP70 mRNA and protein levels in response to HS. (**A**) HSP70 relative mRNA expression in iPSCs from P78S (HHL8^−/−^) and V54L (HHL9^−/−^, HHL10^−/−^) patients and from CTRs (CTR8^−/+^, CTR9^−/+^, and CTR^+/+^) incubated under normal (37 °C) or HS (41 °C) conditions for a period of 4 h. HSP70 mRNA levels were determined by qRT-PCR and normalized to levels of the housekeeping gene GAPDH. Data are presented as mean ± SEM of three independent experiments, each performed in triplicates. Each data set was tested for normal distribution followed by multiple unpaired *t*-tests for comparisons within lines (#). One-way ANOVA with Tukey post hoc analysis for multiple comparisons was used to compare different lines marked (*). #/* *p* < 0.05, *** *p* < 0.001. (**B**) Representative blots of HSP70 and GAPDH in P78S and V54L families under normal (37 °C, left panel) or HS (41 °C, right panel) conditions. Quantification of HSP70 protein levels of undifferentiated iPSCs cultured at 37 °C (**C**) or following four hours of HS at 41 °C (**D**) for all lines normalized to GAPDH. The presented data are from five experiments performed across different passages. Mann–Whitney analysis was conducted for comparison of each patient line and their corresponding CTR from the same family.

The translocation of HSP70 into the nucleus in response to heat shock (HS) has previously been demonstrated to depend on HIKESHI [5]. To test HSP70 expression in iPSCs, we tested its expression at both the mRNA and protein levels. HSP70 mRNA levels were upregulated in response to HS in both the CTR and HHL lines (Figure 5A). Notably, HSP70 mRNA levels exhibited a more pronounced elevation in HHL cells in response to HS (*p*-value < 0.05, one-way ANOVA). These findings suggested an exaggerated response to HS in HHL cells. Western blot analysis showed no significant differences in HSP70 protein levels in response to HS treatment in either the CTR or the HHL lines (Figure 5B–D).

**Figure 6:**
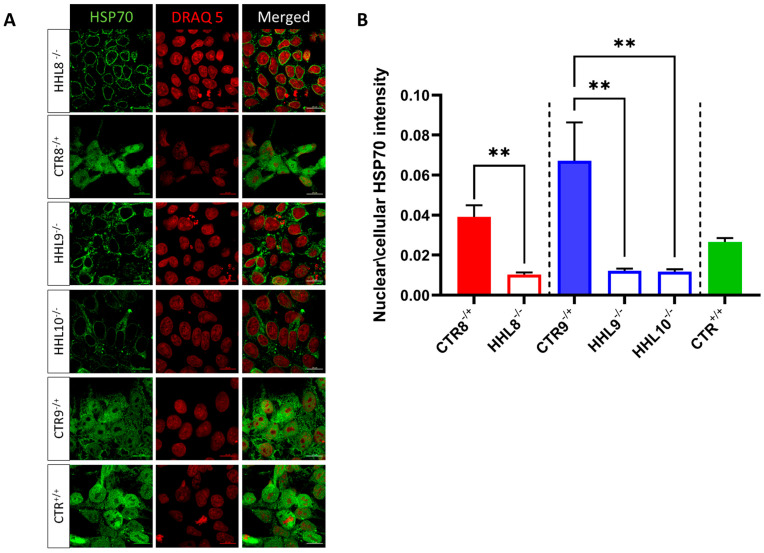
HSP70 nuclear localization following HS treatment. (**A**) iPSCs from HHL patients and CTR were stained for HSP70 (green) and DRAQ5 (red) following HS. Scale bar = 20 μm. (**B**) Quantification of the ratio of the nucleic to cytosolic intensity of the stained HSP70. Values represent mean ± SEM from at least four biological repeats per line. For comparison, one-way ANOVA was performed using PRISM 10 GraphPad software. When *p* values less than 0.05 were received, pairwise comparison between individual groups was used. ** *p* < 0.005.

We next investigated the effect of the pathogenic HIKESHI variants on the cellular localization of HSP70 following HS by immunocytochemistry (ICC) staining using confocal microscopy. Notably, HSP70 expression was observed in cell nuclei in CTR but not in HHL cells following HS (Figure 6A,B). These results indicated that HHL cells had an impaired response to HS and confirmed that HIKESHI was necessary for the translocation of HSP70 to the nucleus.

## 3. Discussion

HHL is a rare neurodegenerative inherited white matter disorder caused by inactivating mutations in the *HIKESHI* gene. HHL patients display early onset progressive spasticity and nystagmus and radiologic features typical for patients with severe hypomyelinating leukodystrophies [15]. In addition, HHL patients are highly vulnerable during febrile illness. In less severe cases, such episodes can lead to deterioration of symptoms. In more severe cases, febrile illness was reported to lead to sudden death, in some instances in association with perimyocarditis [1,2,3]. Indeed, in addition to symptomatic care, clinical management is focused on preventing febrile episodes and early intervention during febrile illness with hemodynamic stabilization and anti-inflammatory medications including high dose steroids depending on the clinical status. Thus, improved therapeutic strategies are needed. Their development is dependent on better understanding of the mechanisms underlying HHL and the availability of robust disease models.

A recurrent pathogenic variant, with high prevalence in the AJ population, was previously identified [1]. A single case of an additional HIKESHI pathogenic variant was identified in a Finnish HHL patient [3], and another novel variant was recently described in an Afghan patient [4]. Our study now identified a novel HIKESHI pathogenic variant. Our clinical investigation suggested that the proband that we described shared clinical similarities and displayed similar diffuse hypomyelination to that of the previously described HHL patients.

HIKESHI is a relatively compact protein of about 22 kDa [8,16], which forms an asymmetrical homodimer through its flexible C-terminal domain (CTD) and linker region, which regulates its binding to HSP70 [10]. Our in silico analysis suggests that unlike the V54L and C4S variants, which are located within the hydrophobic pocket [3], the P78S pathogenic variant residue is located in a distinct region that is involved in the HIKESHI dimerization region. It is therefore plausible that the P78S mutation interferes with dimerization of the asymmetric homodimer, rendering it non-functional.

Despite the high prevalence of heterozygous *HIKESHI* mutation carriers within the AJ population, only a small number of HHL patients were diagnosed and reported to date. Based on that, the calculated disease prevalence was 1:160,000, suggesting that homozygous *HIKESHI* mutations may be embryonically lethal. It is therefore possible that *HIKESHI* pathogenic variants may be the cause of misdiagnosed miscarriages, particularly in the AJ population. The survival of rare cases with pathogenic HIKESHI mutations to adulthood remains a mystery and could be influenced by genetic background or other variables, such as the response to inflammation during specific developmental stages. Interestingly, a *Hikeshi^−/−^* mouse was reported to be lethal at a pre-weaning stage [17,18]. This limits the use of mouse models for studying the role of HIKESHI during embryonic and neonatal development and calls for the development of human-relevant disease models.

We therefore generated and characterized a comprehensive set of iPSCs from HHL patients carrying the homozygous V54L or P78S mutation, as well as from matching heterozygous carrier and unrelated non-carrier healthy CTRs. Our results showed that both V54L and P78S HHL iPSCs expressed CTR-like *HIKESHI* mRNA levels. However, at the protein level the P78S HHL cells had extremely low protein levels, and the V54L cells were HIKESHI depleted following HS. These results suggested that both the V54L and P78S variants may result in exposure of hydrophobic residues, which eventually leads to protein degradation. This is in agreement with a previous study that used an overexpression system to suggest that the pathogenic variants V54L and C4S have low protein levels [19].

Since HIKESHI was previously shown to play a role in the HS response machinery [5,9,16], we tested its expression following HS treatment. Given the critical role of HIKESHI in facilitating HSP70 translocation into the nucleus during HS stress, we examined the expression of HSP70 following HS in iPSCs. While *HSP70* mRNA levels were upregulated in both HHL and control iPSCs in response to HS, the HS-induced mRNA levels were notably higher in the HHL cells. These results confirmed an abnormal and possibly exaggerated HS response in HHL cells, correlating with the sensitivity of HHL patients to febrile illness. At the protein level, HSP70 levels were not affected in either the CTR or the HHL cells. The discrepancy between the elevated *HSP70* mRNA levels and the corresponding protein levels may be attributed to the relatively short time interval between HS exposure and sample collection. Our ICC analyses showed that HS treatment resulted in higher HSP70 nucleus expression in the CTR compared with HHL iPSCs. These results suggested that HIKESHI mediated shuttling of HSP70 into the nucleus and that this translocation was impaired by both the V54L and the P78S pathogenic mutations. It is possible to speculate that cytoplasmic HSP70 functions are retained in HHL cells. The results in the V54L variants are in agreement with previous reports [5,6,9,16]. The effect observed on P78S cells suggested that both variants may impair a common mechanism. Since our results indicated that levels of the P78S variant were markedly decreased, it is possible that insufficient HIKESHI was available to mediate HSP70 translocation to the nucleus. Alternatively, the variant may impair HIKESHI’s ability to bind HSP70. Further investigation will be required to clarify how the P78S variant modulates HSP70 nuclear translocation during the heat shock response.

Overall, the identification of a novel HHL-causing mutation expands the spectrum of mutations associated with HHL, emphasizing the need for further research to fully elucidate the underlying mechanisms of this devastating disorder. The use of HHL iPSCs suggests that HIKESHI may play a role in development, at least in part by mediating HSP70 shuttling into the nucleus. Patient-derived iPSCs provide a valuable platform for investigating disease mechanisms and potential therapeutic approaches in HHL and other related disorders. Future studies using these cells may focus on exploring HIKESHI’s role in disease-relevant tissues, such as specific neural subtypes and oligodendroglia cells [19], as well as cardiac tissue, to gain further insights into its broader functions and implications for human health.

## 4. Materials and Methods

All materials and reagents are described in Supplementary Table S1.

### 4.1. Sample Collection

Skin biopsies of affected individuals with HHL and healthy controls were collected at the TASMC.

### 4.2. Generation of Fibroblasts and Cell Reprogramming

Primary fibroblasts were retrieved from patients’ and CTRs’ skin biopsies. Fibroblasts were cultured in DMEM with 15% fetal bovine serum (FBS) and 1% penicillin-streptomycin and incubated at 37 °C, 5% CO_2_. Reprogramming was performed by harvesting cells and electroporating them with non-integrating episomal vectors using a Neon transfection system (Invitrogen, Waltham, MA, USA). Following that, cells were plated on mouse embryonic fibroblast (MEF)-coated plates and cultured with DMEM with 15% fetal bovine serum (FBS), 5 ng/mL basic fibroblast growth factor (bFGF), and 5 µM ROCK inhibitor. Two days later, media was switched to NutriStem supplemented with 5 ng/mL bFGF, and media was replaced every other day. Sixteen to twenty days later, six colonies with typical iPSC morphology were picked and transferred to new MEF-coated plates and were cultured in NutriStem supplemented with 5 ng/mL bFGF. Three colonies were then picked and manually transferred to Matrigel (Corning, Corning, NY, USA)-coated plates and cultured with NutriStem. Media was replaced daily, and cells were passaged weekly.

### 4.3. iPSC Culture

iPSC lines were cultured in NutriStem hESC XF medium on Matrigel-coated plates. All cells were cultured in standard conditions of 37 °C and 5% CO_2_, in a humidified Eppendorf incubator. Cells were passaged every 3–4 days or when confluence was 85–90% with TryplE Express Enzyme. A mycoplasma evaluation was performed regularly using the PCR mycoplasma detection kit, Life Technologies, Delhi, India.

### 4.4. Heat Shock Treatment

For inducing HS treatment, cells were incubated at 41 °C, 5% CO_2_, for 4 h prior to the various analyses.

### 4.5. Immunocytochemistry Analysis

Cells were initially washed with Dulbecco’s phosphate buffered saline (DPBS) and then fixed with 4% paraformaldehyde (PFA) for 20 min at room temperature. Following fixation, the cells were washed twice with DPBS. The samples were then incubated for 1 h in a blocking buffer consisting of 1% bovine serum albumin (BSA) in DPBS supplemented with 0.1% Triton X-100. Subsequently, the cells were incubated overnight at 4 °C with primary antibodies in the blocking solution. The next day, the samples were washed twice with the blocking solution and incubated for 2 h at room temperature with fluorescently labeled secondary antibodies. For nuclear staining, the cells were incubated with DAPI (1:100, Sigma Aldrich, St. Louis, MO, USA) for 3 min. The primary antibodies were used at the following dilutions: rabbit anti-HSP70 (1:500), mouse anti-OCT3/4 (1:100), mouse anti-TRA-1-60 (1:50), mouse anti-SSEA4 (1:100), rabbit anti-SOX2 (1:100), rabbit anti-NANOG (1:100), rabbit anti-Neurofilament (1:100), rabbit anti-alpha-Fetoprotein (1:1), and rabbit anti-alpha-smooth muscle actin (1:100). The used secondary antibodies were Alexa Fluor 594 donkey anti-rabbit and Alexa Fluor 488 donkey anti-mouse (1:500).

### 4.6. Genotyping

DNA from iPSCs was extracted using the Puregene Cell kit, Hilden, Germany, according to the manufacturer’s instructions. DNA fragments of the region around the mutations were amplified by PCR and Sanger sequenced (see Table 1).

### 4.7. RNA Extraction and Quantitative Real-Time PCR (qRT-PCR)

RNA was extracted from cell pellets of iPSCs by Geneaid Blood/Cell total RNA mini kit, and 1 µg of total RNA was transcribed into cDNA in 20 µL reactions using the qScript cDNA Synthesis Kit, Redwood City, California. TaqMan Probe-Based RQ-PCR analysis was performed using the sequences of TaqMan PCR primers and probes by Thermo Fisher Scientific, Waltham, Massachusetts, and the Fast Advanced Master Mix, according to the manufacturer’s instructions. The sequences of TaqMan PCR primers and probes are presented in Table 2. The TaqMan probe was fluorescent labeled at the 5′ end with 6-carboxyfluorescein (FAM) as the reporter dye and at the 3′ end with NFQ as the quencher dye. TaqMan PCR was performed in 384 well plates with a final volume of 10 µL PCR mixture. Primers and probes were previously titrated to check for amplification efficiency. Amplification and detection were performed in Quantsudio5 and analyzed in Design and Analysis 2.5.1 software.

### 4.8. Western Blot Analyses

Cells were cultured and collected for protein extraction at 80–90% confluence on an ice-cold platform with a lysis buffer containing 0.1% triton (Sigma Aldrich), 1 tablet of cOmplete™ ULTRA Tablets EDTA-free Protease Inhibitor Cocktail, and 1 tablet of PhosSTOP™ Phosphatase inhibitor Cocktail per 10 mL of buffer (Roche, Basel, Switzerland). The cells were incubated with the lysis buffer on ice for 10 min after collection. Next, cell lysates were manually homogenized for 3 min and then centrifuged for 15 min in 15,000 RMP in 4 °C. The total protein in the supernatant was quantified using a Bradford assay with protein dye reagent (Bio-rad, Hercules, CA, USA) using BSA protein as the standard curve. An amount of 30 µg of total protein samples was loaded into a 10% Poly-acrylamide gel (separating gel: 1.5 M TRIS-HCl [pH 9.8] 0.1% SDS; stacking gel: 1 M TRIS-HCl [pH 6.8], 0.1% SDS) in a Mini-PROTEAN^®^ Tetra Cell (Bio-Rad). Samples were stacked through the gel in 100 V for 10 min, separated for 1 h on 125 V in TRIS-GLYCINE running buffer (25 mM TRIS, 192 mM Glycine, 0.1% SDS), and then transferred onto a nitrocellulose membrane (Sartorius, Göttingen, Germany) in a Trans-Blot^®^ SD Semi-Dry transfer cell (Bio-Rad) in 13 V for 30 min (per membrane). Membranes were blocked for 1 h at RT in 5% Skim-milk (Sigma Aldrich) in TBS (20 mM TRIS, 150 mM NaCl). Following the blocking, membranes were incubated over night at 4 °C with primary rabbit anti-HIKESHI polyclonal antibody (1:500, abcam 20524-1-AP) in 5% Skim-milk. Following the primary antibody, membranes were washed with TBST (TBS containing 0.1% Tween-20) for 3 min, followed by 3 washing cycles of TBSx1. Next, the membranes were incubated with Goat anti-rabbit IgG secondary antibody-HRP for 2 h at RT. Afterward, membranes were again washed and moved to chemiluminescence imaging using Westar Supernova in a Fusion Solo X imaging system, Collegien, France. Additional staining for normalization was performed for all membranes using mouse anti-GAPDH (1:1000) and Goat anti-mouse. To test multiple antibodies on the same samples, membranes were stripped of antibodies by washing the membranes with TBST for 3 min. Following TBST, membranes were washed twice with a pre-warmed (55 °C) stripping buffer (200 mM Glycine, 0.1% SDS, 0.2% Tween-20) for 10 min. Next, the membranes were washed with TBST for 3 min followed by 2 rounds of washing with TBS for 3 min. Following membrane stripping, membranes were blocked again for 1 h at RT in 5% Skim-milk and then incubated with primary followed by secondary antibodies.

### 4.9. Imaging

Imaging was performed either using ZEISS LSM 900 Airyscan 2, Jena, Germany, combined widefield confocal microscopy with a super-resolution system or using an EVOS digital color fluorescence microscope system (Invitrogen).

### 4.10. HSP70 Intensity Quantification of Confocal Microscopy Images

To quantify the amount of HSP70 inside and outside the nuclei, the nuclei were initially segmented using Cellpose’s nuclei model [20]. The segmented objects were then imported as regions of interest (ROIs) into Fiji-ImageJ-win64 [21]. For each ROI, both the area and the overlapping HSP70 signal were measured. Additionally, the total area of the image and the total HSP70 signal were quantified to provide comprehensive data for analysis.

### 4.11. Statistical Analysis and Data Visualization

Graph-pad Prism was used to run statistical analyses for qRT-PCR, Western blot, and image analyses. All data were first tested for their normal distribution using the Shapiro–Wilk test. Normally distributed data sets were analyzed using one-way ANOVA with Tukey’s test for multiple comparisons. *T*-tests or multiple *t*-tests were used to analyze paired samples (either the same clone under two treatments or clones derived from the same family). Data sets that were not normally distributed were analyzed using the Mann–Whitney nonparametric test or the Kruskal–Wallis nonparametric test. The results were presented as mean ± SEM. Values *p* < 0.05 were considered significant (* *p* < 0.05, ** *p* < 0.01, *** *p* < 0.001, **** *p* < 0.0001).

## 5. Conclusions

This study identified a novel homozygous pathogenic variant, c.232C>T (P78S), in a patient with HHL, broadening the known mutation spectrum. The P78S and V54L variants resulted in impaired HIKESHI levels, leading to defective HSP70 translocation into the nucleus following HS. Using patient-derived iPSCs, we developed an in vitro model for HHL that demonstrated the critical role of HIKESHI in mediating HSP70 nuclear translocation in undifferentiated iPSCs. This model provided a valuable foundation for future research into HHL pathogenesis and for assessing potential therapeutic strategies.

## Figures and Tables

**Table 1:** Sequences of primers for PCR analysis.

Target	Direction	Primer Sequence 5′-3′
*HIKESHI* Intron 1	Forward	GGCCTCCCACAAAGTGTTGG
*HIKESHI* Intron 2	Reverse	ATTTTTTTTGGCCGGGCGC
*HIKESHI* Intron 1	Forward	CAGAAACTCATAGAACCATTGAACTG
*HIKESHI* Intron 2	Reverse	TATGACTTTTCCCCCTTTAGATTGTG

**Table 2:** Sequences of TaqMan PCR primers.

Gene Name	Primer Sequence 5′-3′	Assay ID
*HIKESHI*	GTCTTAAATCTGGAGAAGGAAGCCA	*Hs01581971_g1*
*HSP70*	TTTTCCGGTTTCTACATGCAGAGAT	*Hs00359163_s1*
*GAPDH*	CGCTGCCAAGGCTGTGGGCAAGGTC	*Hs02786624_g1*

## Data Availability

Accession numbers for the data sets of sequences generated and analyzed for this study are all available in GenBank: PQ773159, PQ773160, PQ773161, PQ773162, PQ773163, and PQ773164. Additional details regarding the analysis and data reported in the study are available upon request by contacting the lead corresponding author, Gad D. Vatine (vatineg@bgu.ac.il).

## Supplementary Materials

The following supporting information can be downloaded at: https://www.mdpi.com/article/10.3390/ijms26136037/s1.

## Abbreviations

The following abbreviations are used in this manuscript:

HHL	Hikeshi-related hypomyelinating leukodystrophy
AJ	Ashkenazi Jewish
CA	Christian Arab
HS	Heat shock
iPSCs	Induced pluripotent stem cells
HSP70	Heat Shock Protein 70
CNS	Central nervous system
V54L	Val54Leu (mutation in *HIKESHI* gene)
P78S	Pro78Ser (mutation in *HIKESHI* gene)
