# Supplementary material for: A Novel Pathogenic Variant Identified in HIKESHI-Related Hypomyelinating Leukodystrophy Disrupts Heat Shock Response in iPSCs
Source: Int J Mol Sci. 2025 Jun 24;26(13):6037. doi: 10.3390/ijms26136037 (PMC12250047; doi:10.3390/ijms26136037)
Supplement: Supplementary file 1 [file ijms-26-06037-s001.zip › ijms-3688973-supplementary.pdf]

Supplementary Figure S1. iPSC characterization - pluripotency

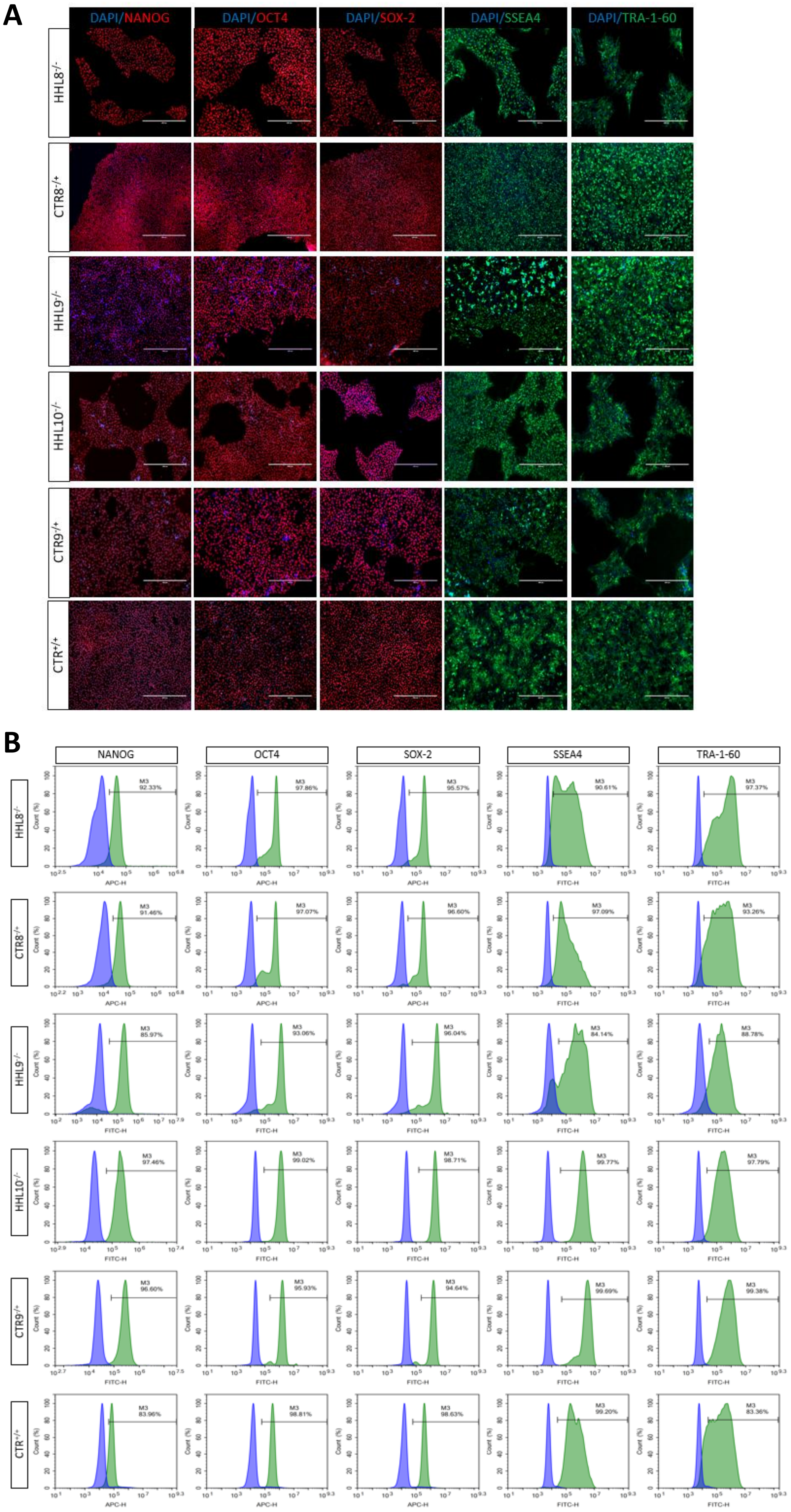

Supplementary Figure S2. Spontaneous differentiation and karyotyping

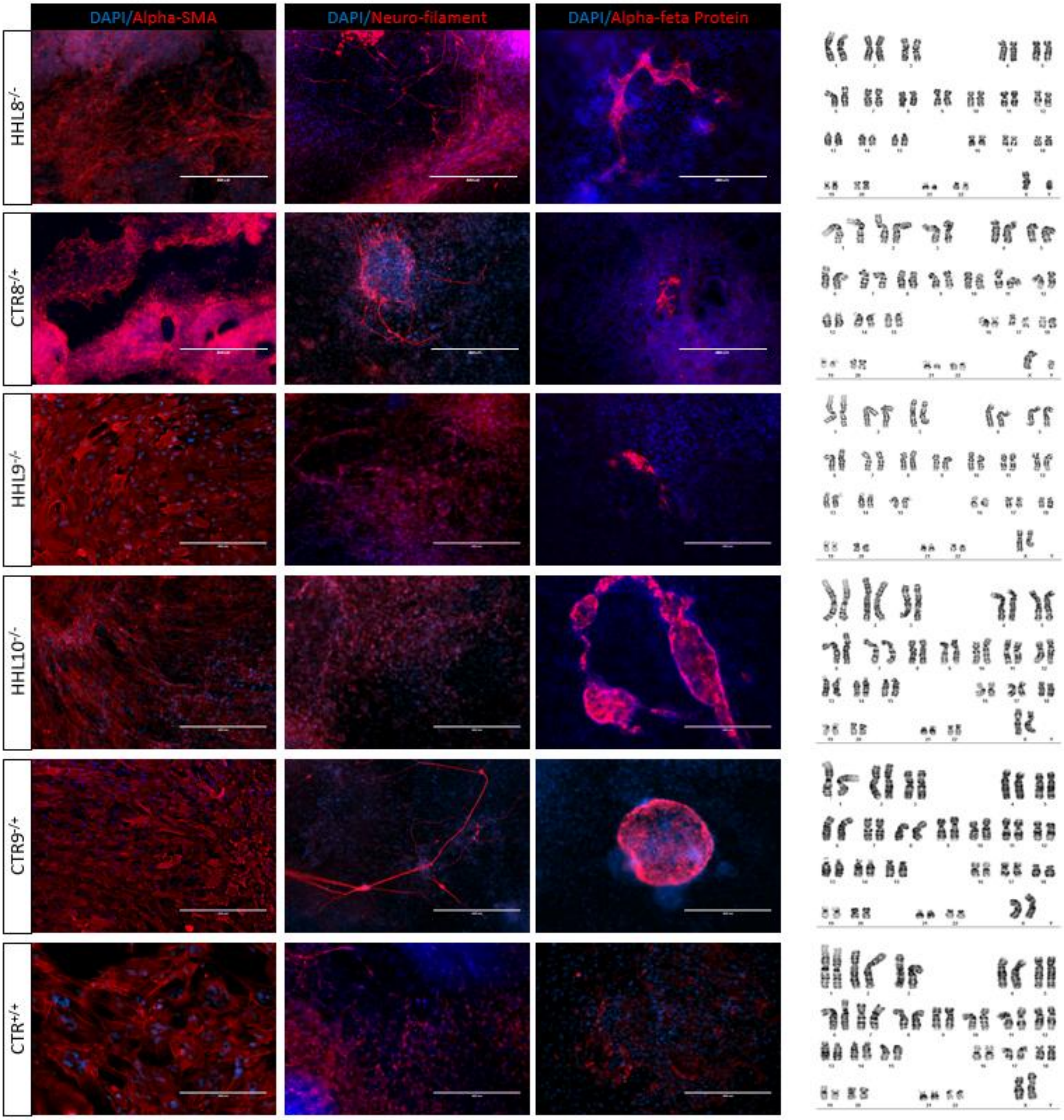

Supplementary Figure S3. Full Western blot membranes

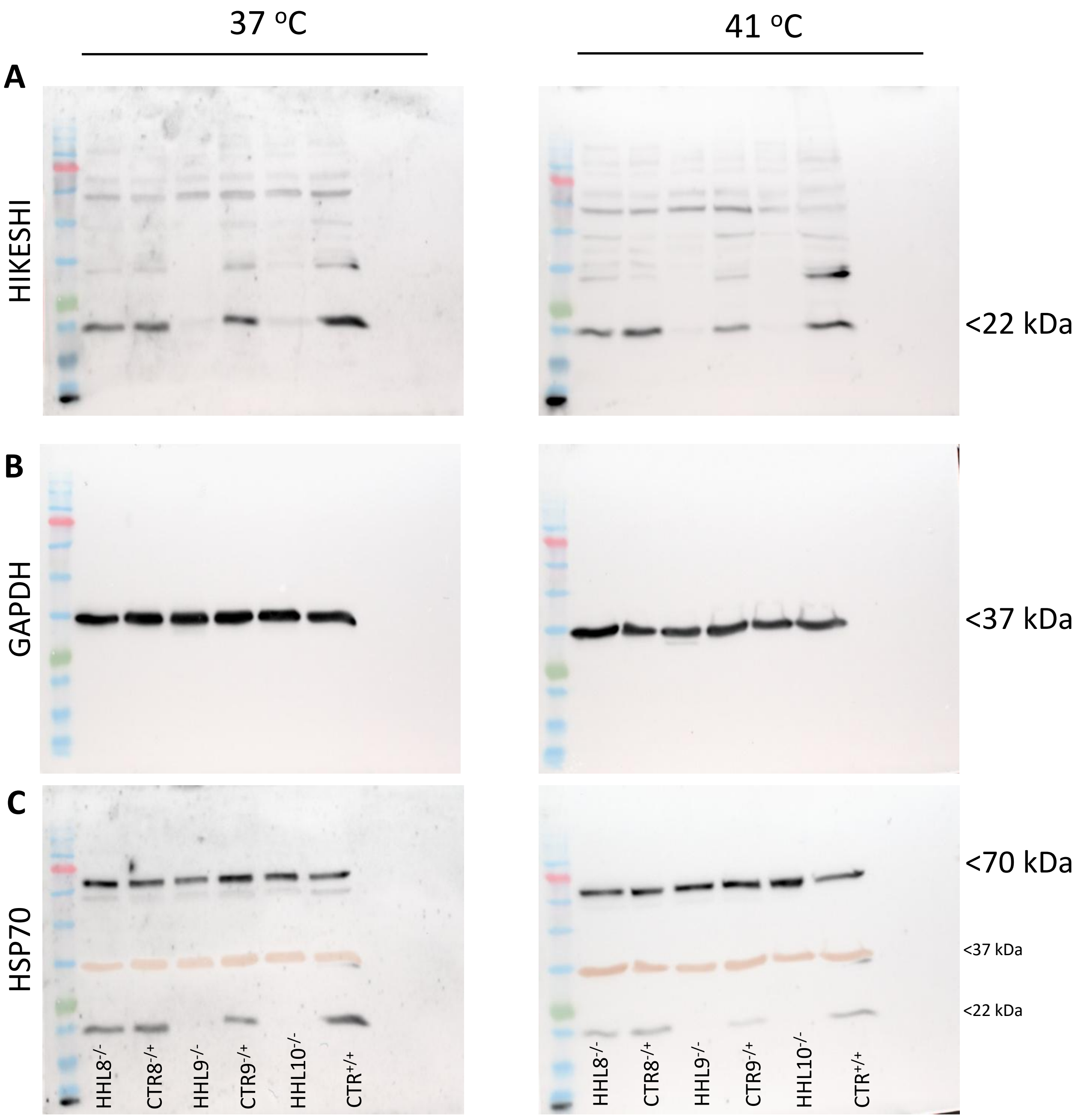

Table S1. Materials and reagents.

REAGENT or RESOURCE	SOURCE	IDENTIFIER
Biological samples		
Skin biopsies of affected individuals with HHL and healthy controls	Patients with a diagnosis of HHL	IRB: 0464-20 TLV & 0586-20-TLV
Cell culture		
Primary Fibroblasts	Skin biopsies of affected individuals with HHL and healthy controls	IRB: 0464-20 TLV & 0586-20-TLV
iPSCs	Primary Fibroblasts	BGU008iHikeshi n1<br>BGU008iHikeshi n2<br>BGU008iHikeshi n3<br>BGU008iHikeshi CTR n4<br>BGU009iHikeshi n1<br>BGU009iHikeshi n2<br>BGU009iHikeshi n3<br>BGU009iHikeshi CTR n2<br>BGU009iHikeshi CTR n3<br>BGU009iHikeshi CTR n6<br>BGU010iHikeshi n4<br>BGU012iPTHS CTR n6<br>BGUiOMENTUM1-1
Bacteria and virus strains		
non-integrating episomal vectors addgene	pCXLE-hOCT3/4-shp53<br>pCXLE-hSK<br>pCXLE-hUL<br>pCXWB-EBNA1<br>pEP4 E02S ET2K	27077<br>27078<br>27080<br>37624<br>20927
Antibodies		
anti-HSP70	Abcam	ab79852
anti-OCT3/4 (C-10)	Santa Cruz Biotechnology	sc-5279
anti-TRA-1-60	R&D	MAB4770

Anti-SSEA-4 (813-70)	Santa Cruz Biotechnology	sc-21704
anti-SOX2	Abcam	AB-ab97959
anti-NANOG	Abcam	AB-ab80892
anti-Neurofilament	Abcam	AB-ab52989
anti-alpha-Fetoprotein	ScyTek	A00058
anti-alpha-smooth muscle actin	Abcam	AB-ab32575
anti-HIKESHI	Abcam	ab202065
anti-HIKESHI	Proteintech	20524-1-AP
anti-GAPDH	Santa Cruz Biotechnology	sc-365062
HRP-conjugated anti-rabbit IgG	GeneTex	GTX213110
HRP-conjugated anti-mouse IgG	GeneTex	GTX213111
Alexa fluor-conjugated 594, donkey anti-rabbit IgG	Jackson Immunoresearch	711-585-152
Alexa fluor-conjugated 488, donkey anti-mouse IgG	Jackson Immunoresearch	715-545-150
Chemicals, peptides, and recombinant proteins		
DMEM Medium	Gibco	C11995500BT
NutriStem	Sartorius	05-100-1A
Foetal Bovine Serum (FBS)	Gibco	C04001-050
Dulbecco's Phosphate Buffered Saline (DPBS)	Sartorius	02-023-1A
Antibiotic-Antimycotic 100X	Biowest	L0010-100
bFGF	Peprotech	100-18B
ROCK inhibitor	Enzo	ALX-270-333
Matrigel	Corning	FAL354230
TryplE Express Enzyme	Gibco	12604021

Bovine Serum Albumin (BSA)	Sigma-Aldrich	A7906
Triton X-100	Sigma Aldrich	T8787
Paraformaldehyde (PFA)	Belgar	32120132
cOmplete Mini	Roche	11836153001
Phos STOP	Roche	04 906 837 001
Bio-Rad Protein Assay	Bio-rad	5000006
FOXP3 Fix/Perm Buffer Set	eBioscience	00-5523-00
Westar Antares	Cyanagen	XLS142,0250
Westar Supernova	Cyanagen	XLS3,0100
Oligonucleotides		
Primers for PCR and Sanger sequenced	Sigma-Aldrich	**Table 1**
TaqMan Probe-Based primers and probes	Thermo Fisher Scientific	**Table 2**
Critical commercial assays		
cDNA Synthesis Kit	Quantabio	95047
Geneaid Blood/Cell total RNA mini kit	Hylabs	RB300
Puregene Cell kit	Qiagen	158845
Fast Advanced Master Mix	Applied Biosystems	4444557
Neon transfection system	Invitrogen	MPK10096
HY-Mycoplasma PCR KIT	Hylabs	KI 5034I
Software		
GraphPad Prism 10.0.0	GraphPad Sofware	https://www.graphpad.com/
ImageJ	Schneider et al.,2012	https://imagej.nih.gov/ij
Adobe Illustrator	Adobe	https://www.adobe.com/

Design and Analysis 2.5.1 software	Thermo Fisher Scientific	https://www.thermofisher.com/il/en/home/technical-resources/software-downloads.html
Instrumentation		
Quantsudio5	Applied Biosystems	A34322
Fusion Solo X imaging system	Vilber	V0.70 9999 9989 2
ZEISS LSM 900 Airyscan 2	ZEISS	
Amis ImageStream Mk II	Amnis, Co	
Deposited data		
BLAST NCBI	blastn	https://www.ncbi.nlm.nih.gov/geo/query/blast.html
EMBL-EBI	Clustal Omega	https://www.ebi.ac.uk/jdispatcher/msa/clustalo
